# Aristocracy of Profession

**Published:** 1882-08

**Authors:** J. A. Robinson

**Affiliations:** Jackson


### ARISTOCRACY OF PROFESSION.

BY J. A. ROBINSON, D.D.S.

It was my privilege to attend the recent meeting of the Indiana State Dental Association ; it was largely attended, some of the best minds in the profession were present, and it was a profitable meeting. Notwithstanding all that can be said in its praise, there was a point of professional weakness in a resolution offered first "forbidding," afterward modified to "requesting dental dealers or their representatives to refrain from mentioning or calling attention to their goods on the floor of the association." From the discussion that followed before its passage it was patent to every one that it was intended to strike Mr. Welch of "Items of Interest," who was present and who might say something in favor of his particular line of goods or his paper. The little petty jealousies that grow out of rivalries among dealers ought never to be brought into an association ; and the resolution was calculated to arouse passions—and persons who have thoughts and passions are oftentimes governed by passions.

Passions are the watch dogs that guard the profession. They picket professions as soldiers do the standing armies. This grows out of the natural tendency of combined associations to arrogate to themselves privileges that others do not possess. It is the weakness of human nature and is manifested in the minds of those who use professions merely for the sake of getting a living or of obtaining wealth, and that is the narrowest use to which a profession can be turned. The usefulness of an institution must always depend upon the breadth and qualities of individual members. The thoughts and suggestions that sparkle in individual minds as they meet together in conventions are *somethings*; they are real entities that perhaps we get only glimpses of while alone, and the various minds when brought into contact are like the combined elements that produce the electric light that lightens the world.

The advantages of these meetings are the same as visiting gardens or going to the woods for new flowers with a botanist; the new form is added to our stock of information, it is part of us, and the form of what we see or hear (for everything is a form in the mind) becomes part of us in memory. If it is good we become more God-like in the world and are greater helps to the association.

Aristocracies belong to the past and are incompatible in republican institutions. Institutions like men should be protected, they should never be guarded. The perturbations caused by introducing new modes into old forms disturbs the tranquility of aristocratic minds that are always surrounded by dreamy visions of rest that are never realized. The aristocratic rest in the days of Louis XIV was the remote cause of the French revolution. In saying this we do not wish to defy public sentiment, but to lead to a higher and better sentiment. If we break old and established notions we must take the consequences that naturally grow out of such a course.

It is easy to float down stream but it takes manly courage to swim against the current. Professional men should never feel that they are too fully occupied to busy themselves with untried notions; they cannot afford it. We must all allow the world has moved along, and we should ask ourselves how much we have helped to move it. Nature, as a rule, does not divulge her secrets without real hard work. If we say we discover by accident, the paths that led to the accident are often long and tiresome. While we may rejoice at our progress there is yet great infirmity in our men and in our materials. The forms of association and society often crush out the free spirit of the age. We hang on to the past with the tenacity of the Jews to their religion. There is no object pertaining to practice or instruments too sacred to be brought before the assembled convention.

If men are to be shut off it will be the end of all advancement and the profession will go back to the days of secrecy that abounded with the early practitioners. Nothing will stimulate the individual as much as talking about his pet practice or his pet instrument. The world has been moved along by the omnipotent power ot talk.

It is better to bear a little egotism than profound silence. When Napoleon entered Moscow in silence he said to his Generals, "Sweeter would be the roar of ten thousand cannons." There is no way of advance so fast as liberty of speech on every new theory or instrument whether it is for sale or a free gift. It is the freedom of individual mind that has raised our profession so rapidly, and given it an acknowledged place in community, and a seat beside the doctor of medicine.

It will not do to destroy the freedom of the God part of man any more than the elements of nature. It is only the Guiteau part that must be destroyed.

O, the slavery of conventionalities! O, the ruts of aristocracy! How they handicap our highest aspirations. All the elements combine in harmony to produce the exquisite blossom and odour of the rose. The latent energies of nature combine with the labor ot man to produce the abundant wheat harvest in Indiana. Our freedom of thought must correspond to our republican institutions. The Austrian Generals complained that Napoleon the First did not fight his battles according to military law, he improvised new methods, and the Austrians were beaten. The whole truth has not arrived, some truths may shine with a meteoric light in certain directions to aid us in our research, but the whole truth has not been reached. In many things, in prosthetic as well as operative practice, in the most excellent papers that were read, and in the most lucid discussions; this meeting was the blossoming of the most perfect flower we have ever witnessed at a dental meeting, and we hope the worm of aristocracy will not be allowed to gnaw at the tap root of the institution and destroy its usefulness.

JACKSON, July 2, 1882.

